# Caregiver burden and its associated factors among primary caregivers of stroke survivors at Amhara regional state tertiary hospitals: a multicenter study

**DOI:** 10.3389/fstro.2023.1226140

**Published:** 2023-09-07

**Authors:** Gebremariam Bekele, Melisew Mekie Yitayal, Yihalem Belete, Yisak Girma, Tesfa Kassa, Yohannes Awoke Assefa, Solomon Gedlu Nigatu, Getachew Azeze Eriku

**Affiliations:** ^1^Department of Physiotherapy, College of Medicine and Health Sciences, Bahir Dar University, Bahir Dar, Ethiopia; ^2^Department of Physiotherapy, School of Medicine, College of Medicine and Health Sciences, University of Gondar, Gondar, Ethiopia; ^3^Department of Occupational Therapy, School of Medicine, College of Medicine and Health Sciences, University of Gondar, Gondar, Ethiopia; ^4^Department of Epidemiology and Biostatistics, Institute of Public Health, College of Medicine and Health Sciences, University of Gondar, Gondar, Ethiopia

**Keywords:** caregiver burden, caregiver strain, primary caregivers, informal caregivers, stroke survivors, Ethiopia

## Abstract

**Background:**

Caregivers of stroke survivors play a crucial role in post-stroke functional recovery and the prevention of complications. Although the situation is incredibly stressful and intimidating and the caregiver burden is high, there is little evidence in the local Ethiopian context of the extent of the burden among caregivers of stroke survivors. Therefore, the aim of this study was to assess the level of caregiver burden and its associated factors among primary caregivers of stroke survivors in Ethiopia.

**Methods:**

A cross-sectional study was conducted in Amhara regional state tertiary hospitals from April to June 2022. A standardized questionnaire was used to record factors associated with caregiver burden, including sociodemographic, clinical, and care situation factors. The Zarit caregiver burden interview (short form) was used to assess the level of caregiver burden. A systematic random sampling method was employed to select the study participants. Multinomial logistic regression was employed to identify the potential factors associated with the level of caregiver burden.

**Results:**

The overall prevalence burden among primary caregivers of stroke survivors was 67%. 61.1% had a mild to moderate burden, while 5.9% had a severe burden. In multivariable multinomial logistic regression analysis, sex, household income, duration of care, and duration of caregiving hours per day were factors significantly associated with the level of burden among caregivers of stroke survivors.

**Conclusions:**

Being female, having a low household income, caring for more than 3 months, and caring for more than 6 h per day were factors significantly associated with the burdens of the primary caregivers of stroke survivors. It is better: health care providers must recognize and screen for burdens and provide special attention.

## 1. Introduction

Stroke is the second leading cause of death globally and the third leading cause of disability, and the burden is expected to be higher in low- and middle-income countries (Han et al., 2017; Krishnamurthi et al., 2020). Lifelong disability, language, and communication problems, incontinence, and behavioral changes after stroke are the main reasons for long-term rehabilitation and extensive family support for stroke survivors (Le Danseur, 2020; Owolabi et al., 2021).

A caregiver is someone who lives with a stroke survivor and is most closely involved in physical care, providing social and psychological support, and coping with the disease in general (Liu et al., 2020). Caregivers of stroke survivors play a vital role in facilitating functional recovery and preventing stroke sequelae. Caregiving is a stressful and intimidating situation due to the debilitating and chronic nature of the disease, which requires long-term care starting from the healthcare facility to home-based care (Jaracz et al., 2014; Imarhiagbe et al., 2017). The decrease in death rates globally in recent decades (Katan and Luft, 2018) due to medical advances has resulted in an increase in the number of stroke survivors with varied degrees of functional limitations and disabilities that will last a lifetime.

Stroke has a negative impact on the physical, emotional, social, and economic wellbeing of caregivers (Karahan et al., 2014; Hu et al., 2018; Owolabi et al., 2021; Gururaj et al., 2022), which significantly affects the health outcomes and quality of life of stroke survivors (Adelman et al., 2014). The Evidence has suggested that 25–46% of caregivers experience significant distress during the first 6 months of care in the transition from facility-based to home-based care (Blake et al., 2003; Tooth et al., 2005; Van Exel et al., 2005; Ilse et al., 2008), which is often associated with disease severity, depressive and anxiety symptoms, sex, duration and intensity of care, and degree of disability of stroke patients (Blake et al., 2003; Choi-Kwon et al., 2005; Ilse et al., 2008; Bhattacharjee et al., 2012; Gbiri et al., 2015; Jaracz et al., 2015; Kumar et al., 2016).

Caregivers of stroke survivors in resource-limited countries bear a greater burden than those in developed countries. Poor economic status and health care services could be possible reasons for the increased caregiver burden in a resource-limited setting. Shifting responsibility to health professionals, families, and available community-based rehabilitation providers is a cost-effective strategy to improve the access and quality of post-stroke rehabilitation in resource-constrained areas, particularly in Ethiopia (Gururaj et al., 2022; Kayola et al., 2023). Caregivers are therefore encouraged to be actively engaged in post-stroke rehabilitation with the aim of preventing complications and improving the quality of life of the survivors. Caregivers, often friends and family members, play an important role in the rehabilitation of stroke patients, supporting them and preparing them for discharge (Lui et al., 2012; Karahan et al., 2014).

Different factors, such as sociodemographic factors, clinical factors, and other possible factors, could contribute to the perceived burdens of the caregivers. Identifying these factors is important for improving the medical care and rehabilitation of stroke survivors, but in the local Ethiopian context, there is little evidence regarding the magnitude of the caregiver burden and the predictor factors among stroke survivors. We therefore strongly believe that it is relevant to assess the burdens of caregivers and related factors in the Ethiopian context. Therefore, this study aimed to assess the level of caregiver burden and its associated factors among primary caregivers of stroke survivors in Ethiopia.

## 2. Method

### 2.1. Study design, setting, and participants

An institution-based cross-sectional study was conducted among primary caregivers of stroke survivors in four tertiary hospitals in Amhara regional state from April to June, 2023.

The selected hospitals were the University of Gondar, Felege Hiwot, Tibebe Ghion, and Dessie specialized hospitals. Those selected hospitals serve as major referral centers for the Amhara and neighboring regions such as Tigray, Afar, and Benishangul Gumez. Each hospital serves a population of 3.5–5 million.

The source population was all primary caregivers of stroke survivors who attend medical wards and outpatient physiotherapy clinics in selected tertiary hospitals.

Primary caregivers who have been providing care for at least 2 weeks and are aged 18 and above were included in the study.

The caregivers of stroke survivors who are mentally challenged and have difficulty performing activities of daily living due to underlying medical conditions and disabilities were excluded from the study.

### 2.2. Sample size determination and sampling procedures

The sample size was determined by using a single population proportion formula with a 50% prevalence of caregiver burden, a 95% confidence interval, and a 5% margin of error. Finally, a sample of 424 was obtained by adding a 10% non-response rate.


$$\text{ = }\frac{({\text{Za}}_{\text{/2}}){\text{2}}^{*}\text{P}\left(\text{1 - P}\right)}{{\text{d}}^{\text{2}}}\text{=}\frac{\left(\text{1}.\text{96}\right){\text{2}}^{*}\text{0}.\text{5}\left(\text{1 - 0}.\text{5}\right)}{{\left(\text{0}.\text{05}\right)}^{\text{2}}}\text{ = 384}.\text{16}\sim \text{385}$$

Where n-Samples, P-prevalence, d-margin of error

The study participants were proportionally allocated to each hospital based on the report obtained from each hospital. According to the reports of the hospital, at least 65–80 stroke patients were admitted to each hospital. Taking this into consideration, the average number of primary caregivers admitted to UOGSH, TGSH, FHSH, and DSH per 3 months was 218; 207; 228; and 218, respectively. Therefore, during the 3-month data collection period, 792 caregivers of stroke survivors visited the selected hospitals. Thereafter, the skipping interval k^th^ was calculated by dividing the 3-month report by the calculated sample size (870/424), which resulted in 2. Every second caregiver was enrolled from each hospital, with the first one selected randomly. Finally, systematic random sampling techniques were employed to select all eligible study participants. The participants included from UOGSH, TGSH, FHSH, and DSH were 106, 101, 111, and 106, respectively.

### 2.3. Data collection methods and tool

The data was collected using an interviewer-administered questionnaire. The data were collected by four physiotherapists (one for each hospital) who were properly trained for 1 day about the data collection tool, ways of approaching the participants, and how to obtain consent before the data collection.

The data collection tool consists of three sections: the sociodemographic characteristics, the clinical and care situation characteristics, and the Zarit burden interview short form (ZBI-12). The sociodemographic characteristics such as sex, age, educational level, occupation, place of residence, income, and relationships with the stroke survivors. The clinical and care situation characteristics include duration of care, duration of caregiving, time spent on caregiving, having depression, and anxiety symptoms.

The ZBI-12 is a self-administrated tool adapted to measure the subjective stress and perceived social and emotional burdens of the caregivers of stroke survivors. The ZBI-12 consists of 12 items that reflect issues about health, social life, emotional wellbeing, personal life, and interpersonal relationships. It has a total score of 48 on a 5-point Likert scale ranging from 0 to 4 (0 = never, 1 = rarely, 2 = sometimes, 3 = frequently, and nearly always). An increase in the total score indicated a corresponding increase in caregiver burden. Based on the total ZBI-12 scores, the burden level was classified as follows: 0–1 indicates no to minimal burden, 10–20 indicates mild to moderate burden, and a score >20 indicates high to severe burden (Imarhiagbe et al., 2017).

The primary caregiver was defined as the person (family or non-family member) who spent the majority of their time providing daily care to stroke survivors or the person who took on the majority of caregiving tasks (Akosile et al., 2018).

Depression in caregivers was measured using the PHQ-9 depression assessment tool. Those caregivers who scored ≥ 10 were considered to have positive depression symptoms (Achilike et al., 2020).

### 2.4. Data quality control

The questionnaire was first prepared in English, then translated into Amharic and back to English to maintain its consistency. The 1-day training was given by the principal investigators to data collectors (one physiotherapist in each hospital) and supervisors about the purpose of the study and data collection procedures. A pretest was done on 5% of the sample size at Debre tabor referral hospital to check the response and language clarity. After obtaining informed consent, trained data collectors interviewed the study participants. Primary caregivers of stroke survivors willing to participate in the study were screened using the inclusion and exclusion criteria. Participants who were eligible were approached individually and given verbal instructions to choose the best option that they considered appropriate in the questionnaire. Participants took, on average, 25 min to complete the questionnaire.

The readjustment of inconsistent and inaccurate data was done accordingly. The completeness and consistency of the questionnaire were checked daily by the supervisor and principal instigator.

### 2.5. Data processing and analysis

The data were analyzed using IBM SPSS 23.0 statistical software. Descriptive statistics, including frequency and percentage, were used to summarize the data. In order to assess the potential association of the ordinally scaled variables of the caregiver's burden with independent variables, ordinal logistic regression was considered. However, since the goodness-of-fit and proportional lines assumptions were violated, we decided to use the multinomial logistic regression model. Multinomial logistic regression analysis was employed to determine the relationship between the key explanatory variables of interest and the outcome variables of interest (caregiver burden) while controlling the confounding variables. For the outcome variable, “no to minimal burden” was chosen as the reference group for the multivariable logistic regression model. Variables with a *p*-value of 0.25 were considered potential candidates for the final multivariable logistic regression analysis. Multivariable multinomial logistic regression was used to show the association between the dependent and independent variables. The association was expressed in odds ratios (ORs) and with corresponding 95% confidence intervals (CIs).

## 3. Ethical consideration and consent to participate

The ethical clearance was obtained from the ethical review committee of the school of Medicine under the delegation of the ethical review board of the University of Gondar with reference number SOM/1546/2022. After the ethical clearance was received, an official letter of support was received from the University of Gondar and sent to the clinical directors of the selected hospitals. All the study participants were informed of the purpose of the study, the potential indirect benefits, and their right to refuse. Before enrollment, all the study participants provided written informed consent. The confidentiality of the information was assured throughout the data collection.

## 4. Result

### 4.1. Sociodemographic characteristics of the primary caregivers of the stroke survivors

A total of 424 participants with a mean age of 40.2406 (SD = 10.95) years were enrolled in this study. More than half were female (54.2%) and under the age of 40 (58.5%). Nearly two-thirds of the participants were currently married (65.3%) and urban residents (69.1%). The relationship between the caregivers and the stroke patients is as follows: 37.2 were spouses, 30.2 were sons or daughters, and 32.5 were siblings (see Table 1).

**Table 1:** Sociodemographic characteristics of primary caregivers of stroke survivors (*n* = 424).

**Variables**	**Category**	**Frequency (%)**
Age	≤ 40	58.5
	>40	41.5
Sex	Male	45.8
	Female	54.2
Marital status	Married	65.3
	Unmarried	34.7
Residence	Urban	69.1
	Rural	30.9
Educational level	No formal education	21.7
	Primary	18.2
	Secondary	28.1
	Diploma and above	32.1
Occupation	Governmental employee	24.3
	Non-government	16.5
	Farmer	31.4
	Merchant	27.80
Family income	≤ 3,000	38.2
	3,001–6000	37
	>6,000	24.8
Relationship	Sibling	32.5
	Son/daughter	30.2
	Spouse	37.3

### 4.2. Clinical and care situation factors of the care situation of primary caregivers of stroke survivors

From the total numbers of study participants, more than half of them provided care for more than 6 months, and almost three fourths and nearly half of the participants reported symptoms of depression and anxiety, respectively (see Table 2).

**Table 2:** Care situation and clinical of informal characteristics caregivers of stroke survivors (*n* = 424).

	**Category**	**Frequency (%)**
Duration of stroke	≤ 3 months	35.6
	4–6 months	11.6
	>6 months	52.8
Duration of caregiving	≤ 3 months	36.8
	4–6 months	11.3
	>6 months	51.9
Caregiving hours per day	≤ 4	32.3
	5–7	45.8
	≥8	21.9
Depression	Yes	65.6
	No	34.4
Anxiety	Yes	48.8
	No	51.2

### 4.3. Factors associated with caregiver burden in multinomial logistic regression

The caregiver's burden was significantly associated with gender, age, marital status, occupation, family income, relationship, duration of care, and caregiving times. In multivariable logistic regression analysis, sex, family income, duration of care, and time spent on caregiving were significantly associated with the burden of the caregivers of the stroke survivors. Female caregivers were nearly three times more likely to develop mild to moderate and high burdens than male caregivers, respectively. Caregivers with a monthly income of less than ETB 3,000 were three times more likely to develop a mild to moderate burden than those with a monthly income of ETB 6,000. In contrast to this, caregivers with a monthly income of ETB 3,000–6,000 were 0.3 and 0.375 times less likely to have a mild to moderate and high burden, respectively, than those with more than ETB 6,000.

Primary caregivers who have been providing care for more than 3 months are nearly 1.2 and 0.243 times more likely to experience mild to moderate and high burden, respectively, as compared to primary caregivers who have been providing care for <3 months.

Caregivers who have spent more than 6 h per day are nearly four and three times more likely to experience mild, moderate, and high burdens, respectively, as compared to primary caregivers who have spent <6 h per day (see Table 3).

**Table 3:** Factors associated with mild to moderate and high caregiver burden of stroke survivors.

**Variables**	**Category**	**Mild to moderate**		**High burden**	
		***p***	**AOR (95%)**	***P***	**AOR (95%)**
Sex	Female	0.001	2.5 (1.49, 4.2)	0.006	4.35 (1.52, 12.48)
	Male	1	Ref	–	Ref
Age	≤ 40	1	Ref		Ref
	>40	0.661	1.14 (0.642, 2.103)	0.385	1.6 (0.57, 4.4)
Marital status	Married	0.376	0.8 (0.418, 1.39)	0.918	1.1(0.313, 3.637)
	Unmarried	1	Ref	1	Ref
Occupation	Governmental employee	0.344	0.7 (0.343, 1.425)	0.404	0.6 (0.14, 2.23)
	Private employed	0.569	1.3 (0.557, 2.9)	0.198	0.3(0.04, 1.99)
	Housewife	0.0.762	1.1 (0.565, 2.178)	0.928	1.06 (0.28, 4.02)
	Farmer	1	Ref	–	Ref
Family monthly income	≤ 3,000	0.004	2.8 (1.385, 5.713)	0.056	0.112 (0.012, 1.055)
	3,001–6000	0.008	0.3 (0.237, 0.803)	0.012	0.28 (0.099, 0.75)^*^
	>6,000	1	Ref	1	Ref
Relationship	Spouse	0.054	1.95 (0.989, 3.836)	0.225	2.35 (0.59, 9.33)
	Son/daughter	0.64	1.2 (0.642, 2.144)	0.458	1.63 (0.45, 5.95)
	Sibling	1	Ref	1	Ref
Duration of care	≤ 3 months	Ref	Ref	1	Ref
	>3 months	0.493	1.2 (0.72, 1.975)	0.007	0.24 (0.085, 0.68)^*^
Time spent hours	≤ 6	Ref	Ref	1	Ref
	>6	0.000	3.7 (2.036, 6.775)	0.049	2.94 (1.007, 8.58)^*^

* Significant at *p-*value ≤ 0.05,

^**^ significant at *p-*value ≤ 0.001.

## 5. Discussion

The study was conducted among caregivers of stroke survivors in tertiary hospitals in Amhara Regional State. The majority of the study participants in this study were female and married, which is comparable with the study conducted in Asia (Roopchand-Martin and Creary-Yan, 2014; Ahmad Zubaidi et al., 2020; Wang et al., 2021; Liu et al., 2022). More than half of the study participants have been providing care for more than 6 months.

In our study, 67% (284) of primary caregivers experienced a burden from care, with a high proportion experiencing a mild to moderate burden (61.1%) followed by a high burden (5.9%). The finding is consistent with that of studies conducted in China (Cao et al., 2022) and India (Menon et al., 2017) that revealed 67.1 and 65% of the participants were experiencing varying degrees of burden, respectively. But lower than the study conducted in Nigeria (Akosile et al., 2018), where 96.7% of caregivers reported having a high burden. The study was conducted in Nigeria in an acute care setting with a small sample size, and a different outcome measure, the caregiver strain index, was used, which could explain the differences in findings.

On the contrary, our findings are higher than those of a study conducted in Texas, USA (Achilike et al., 2020), which found that 51% of caregivers experienced burden, with 34% experiencing mild to moderate burden and 17% experiencing moderate to high burden, and moderate to severe burden. The variation in the finding could be due to the socioeconomic, cultural, and lifestyle differences between Ethiopia and the United States. For instance, all the study participants in the USA had completed at least their high school education level. In another study conducted in Poland nearly half of the participants, 47% of the stroke survivors experienced severe or moderate strain (Jaracz et al., 2014). The possible reasons for the variation in the results could be the stools used to assess the caregiver burden, the sample size, or the caregiving support system in Europe.

In a multivariable multinomial logistic regression analysis, sex, family income status, relationship, caregiving duration, and time spent per day were significantly associated with the burden of the primary caregivers of the stroke survivors.

Although different studies have shown that age has a significant association with the level of caregiver burden, there was no significant association found in our study. The reason for this could be that almost more than half of the study participants were under the age of 40, and young caregivers felt less burdened because they had a better understanding of the needs of the patients. They are able to deal with challenging situations and adjust to their new function by creating a variety of strategies for coping.

Females were 2,503 and 4,352 times more likely than males to develop mild to moderate and high burdens, respectively, which is consistent with the findings of the studies conducted in India (Menon et al., 2017; Kumar et al., 2022) and Singapore (Wang et al., 2021). The possible explanation could be that female caregivers spend more caregiving hours and are engaged in various household activities due to cultural and moral obligations, which may increase the burden on female caregivers compared to their male counterparts (Pinquart and Sörensen, 2006; Casado-Mejía and Ruiz-Arias, 2016). Additionally, female spouses of stroke survivors harm their psychological wellbeing. Male spouses, on the other hand, have fewer emotional contacts in their social network (Larson et al., 2008). Another study conducted in Iran found that male caregivers were more likely to adopt coping strategies than their female counterparts (Kazemi et al., 2021). In Ethiopian culture, males are the head of the family, while females generally look after the family's wellbeing at home, and women themselves believe that caregiving is their role, and is expected of them by the family as well as the community members.

In this study, caregivers with an average monthly household income of <3,000 ETB were 2.8 times more likely to develop mild to moderate burden than those with a monthly household income of more than 6,000 ETB. The finding was supported by the study conducted in Nepal, which showed that caregivers with a monthly household income of more than 20,000 rupees had a high mean burden score (Sinha et al., 2022). Another similar study conducted in Nepal also showed that financial stress is a significant contributor to the high caregiver burden (Kumar et al., 2022). The possible explanation could be that stroke is the common cause of disability and morbidity (Wafa et al., 2020) and the cost of stroke care is very expensive, which could be the cause of the survivors' and caregivers' economic dependency, resulting in economic and psychological stress. However, caregivers who have an average monthly income of 3,000 to 6,000 ETB were nearly 0.3 times less likely to have both a mild to moderate burden and a high burden as compared to those who have more than 6,000 ETB.

Our study found that caregivers who spent more than 6 h per day were 3.714 and 2.94 times more likely to develop mild to moderate and high burden, respectively, than those who cared for 6 h or less per day, which is similar to the study done in India (Bhattacharjee et al., 2012; Kumar et al., 2022), which found that long caregiving hours were factors increasing caregiver stress. Another study conducted in Nepal on caregivers of patients with spinal cord injuries also revealed that caregivers who provide care for more than 12 h have a high risk of developing high burden (Sinha et al., 2022). Another study done n caregivers of Alzheimer's disease. Yu et al. (2015) also showed that the number of care hours spent per day has a direct relationship with caregiver burden. According to the According to the wear-and-tear hypothesis, the longer caregiving is sustained, the more the caregivers subjective wellbeing declines (Pinquart and Sörensen, 2003).

The study in Malaysia also supported the idea that long-spent care hours increase the risk of developing burden (Ahmad Zubaidi et al., 2020). Another study also found that spending more caring hours with the stroke survivors' harms caregiver's psychological wellbeing and limits their flexibility in caring for the patients. Caregivers were also stressed about balancing work and family life, so they may feel overburdened (Gajraj-Singh, 2011). On the other hand, the study done in Pakistan (Ain et al., 2014) showed that as the duration of care increases, it is significantly associated with a decrease in sleep disturbance, which could be a contributing factor to the development of caregiver burden. Not only for caregivers of stroke survivors, providing prolonged hours per day increases the likelihood of having high caregiver burdens for caregivers of individuals with different conditions like physical disability (Yue et al., 2022), spinal cord injury (Sinha et al., 2022), Alzheimer's disease and cancer etc. Spending more time with stroke survivors may cause caregivers to become physically and mentally frustrated, limiting their ability to care for patients. Our caregivers were also concerned about balancing work and family life; they may have felt a heavy burden.

Caregivers who have been providing care for more than 3 months are 1.2 and 0.24 times more likely to develop mild to the moderate and high burden, respectively, as compared to those who have been caring for <3 months, following existing literature (Visser-Meily et al., 2009; Jaracz et al., 2015; Zhu and Jiang, 2018), which reported that the level of burden decreased over time and the proportion of caregivers with high burden decreased as well. The likely explanation could be that, in the first 3 months, stroke patients' neurological and functional recovery is comparatively high, resulting in minimal support for activities of daily living and partial independence (Lee et al., 2022).

## 6. Strength and limitation

To the best of the author‘s knowledge, this is the first study conducted on the burden among the caregivers of stroke survivors in Ethiopia.

The causal relationship interpretation of the finding could not be established due to the nature of the cross-sectional study. As a result, further prospective studies were needed to establish the casual relationships.

Additionally, the different instruments used to assess the caregiver's burden make it difficult to make reliable comparisons between the various studies.

The other limitation is that we did not include the coping strategies and other possible influencing factors of the caregiver's burden, such as the type of stroke, the extent or severity of the stroke.

## 7. Conclusion

The results of our study showed that primary caregivers experienced a high burden of care which is associated with sex, family monthly income, time spent caring, and duration of caregiving, which were significantly associated with mild to moderate and moderate to severe burdens for caregivers of stroke survivors.

It's better that health care providers in medical and physiotherapy outpatient treatment facilities recognize and screen for burdens and give special attention to caregivers who are female, have low family income, care for more than 6 h per day, and have provided care for more than 3 months to reduce the risk of developing burdens.

## Data availability statement

The original contributions presented in the study are included in the article/supplementary material, further inquiries can be directed to the corresponding author.

## Ethics statement

The studies involving human participants were reviewed and approved by University of Gondar. The patients/participants provided their written informed consent to participate in this study.

## Author contributions

GB and GE were involved in the conception, design, and writing the first draft. GB, GE, and SN were involved with the interpretation and analysis. MY, YB, YG, and YA were involved in revising the final draft. All authors read and approved the final manuscript.

## References

[B1] Achilike S. Beauchamp J. E. Cron S. G. Okpala M. Payen S. S. Baldridge L.. (2020). Caregiver burden and associated factors among informal caregivers of stroke survivors. J. Neurosci. Nurs. 52, 277–283. 10.1097/JNN.0000000000000552 33156591

[B2] Adelman R. D. Tmanova L. L. Delgado D. Dion S. Lachs M. S. (2014). Caregiver burden: a clinical review. JAMA 311, 1052–1060. 10.1001/jama.2014.304 24618967

[B3] Ahmad Zubaidi Z. S. Ariffin F. Oun C. T. C. Katiman D. (2020). Caregiver burden among informal caregivers in the largest specialized palliative care unit in Malaysia: a cross sectional study. BMC Palliat. Care 19, 186. 10.1186/s12904-020-00691-1 33292214 PMC7722979

[B4] Ain Q. U. Dar N. Z. Ahmad A. Munzar S. Yousafzai A. W. (2014). Caregiver stress in stroke survivor: data from a tertiary care hospital -a cross sectional survey. BMC Psychol. 2, 49. 10.1186/s40359-014-0049-9 25520808 PMC4266982

[B5] Akosile C. O. Banjo T. O. Okoye E. C. Ibikunle P. O. Odole A. C. (2018). Informal caregiving burden and perceived social support in an acute stroke care facility. Health Qual. Life Outcomes 16, 57. 10.1186/s12955-018-0885-z 29622011 PMC5887210

[B6] Bhattacharjee M. Vairale J. Gawali K. Dalal P. M. (2012). Factors affecting burden on caregivers of stroke survivors: population-based study in Mumbai (India). Ann. Indian Acad. Neurol. 15, 113–119. 10.4103/0972-2327.94994 22566724 PMC3345587

[B7] Blake H. Lincoln N. B. Clarke D. D. (2003). Caregiver strain in spouses of stroke patients. Clin. Rehabil. 17, 312–317. 10.1191/0269215503cr613oa 12735539

[B8] Cao L. L. Tang Y. F. Xia Y. Q. Wei J. H. Li G. R. Mu X. M.. (2022). A survey of caregiver burden for stroke survivors in non-teaching hospitals in Western China. Medicine 101, e31153. 10.1097/MD.0000000000031153 36550813 PMC9771191

[B9] Casado-Mejía R. Ruiz-Arias E. (2016). Influence of gender and care strategy in family caregivers' strain: a cross-sectional study. J. Nurs. Scholar. 48, 587–597. 10.1111/jnu.12256 27737509

[B10] Choi-Kwon S. Kim H.-S. Kwon S. U. Kim J. S. (2005). Factors affecting the burden on caregivers of stroke survivors in South Korea. Arch. Phys. Med. Rehabil. 86, 1043–1048. 10.1016/j.apmr.2004.09.013 15895355

[B11] Gajraj-Singh P. (2011). Psychological impact and the burden of caregiving for persons with spinal cord injury (SCI) living in the community in Fiji. Spinal Cord 49, 928–934. 10.1038/sc.2011.15 21383762

[B12] Gbiri C. A. Olawale O. A. Isaac S. O. (2015). Stroke management: Informal caregivers' burdens and strians of caring for stroke survivors. Ann. Phys. Rehabil. Med. 58, 98–103. 10.1016/j.rehab.2014.09.017 25752228

[B13] Gururaj S. Bird M. L. Borschmann K. Eng J. J. Watkins C. L. Walker M. F.. (2022). Evidence-based stroke rehabilitation: do priorities for practice change and feasibility of implementation vary across high income, upper and lower-middle income countries? Disabil. Rehabil. 44, 4611–4618. 10.1080/09638288.2021.1910737 33849357

[B14] Han Y. Liu Y. Zhang X. Tam W. Mao J. Lopez V.. (2017). Chinese family caregivers of stroke survivors: determinants of caregiving burden within the first six months. J. Clin. Nurs. 26, 4558–4566. 10.1111/jocn.13793 28252843

[B15] Hu P. Yang Q. Kong L. Hu L. Zeng L. (2018). Relationship between the anxiety/depression and care burden of the major caregiver of stroke patients. Medicine 97, e12638. 10.1097/MD.0000000000012638 30290641 PMC6200450

[B16] Ilse I. B. Feys H. De Wit L. Putman K. De Weerdt W. (2008). Caregivers' strain: prevalence and determinants in the first six months after stroke. Disabil. Rehabil. 30, 523–530. 10.1080/09638280701355645 18365864

[B17] Imarhiagbe F. A. Asemota A. U. Oripelaye B. A. Akpekpe J. E. Owolabi A. A. Abidakun A. O.. (2017). Burden of informal caregivers of stroke survivors: validation of the Zarit burden interview in an African population. Ann. Afr. Med. 16, 46–51. 10.4103/aam.aam_213_16 28469116 PMC5452708

[B18] Jaracz K. Grabowska-Fudala B. Górna K. Jaracz J. Moczko J. Kozubski W.. (2015). Burden in caregivers of long-term stroke survivors: prevalence and determinants at 6 months and 5 years after stroke. Patient Educ. Couns. 98, 1011–1016. 10.1016/j.pec.2015.04.008 25952926

[B19] Jaracz K. Grabowska-Fudala B. Górna K. Kozubski W. (2014). Caregiving burden and its determinants in Polish caregivers of stroke survivors. Arch. Med. Sci. 10, 941–950. 10.5114/aoms.2014.46214 25395945 PMC4223139

[B20] Karahan A. Y. Kucuksen S. Yilmaz H. Salli A. Gungor T. Sahin M.. (2014). Effects of rehabilitation services on anxiety, depression, care-giving burden and perceived social support of stroke caregivers. Acta Med. 57, 68–72. 10.14712/18059694.2014.42 25257153

[B21] Katan M. Luft A. (2018). Global burden of stroke. Semin. Neurol. 38, 208–211. 10.1055/s-0038-1649503 29791947

[B22] Kayola G. Mataa M. M. Asukile M. Chishimba L. Chomba M. Mortel D.. (2023). Stroke rehabilitation in low- and middle-income countries: challenges and opportunities. Am. J. Phys. Med. Rehabil. 102(2S Suppl. 1):S24–S32. 10.1097/PHM.0000000000002128 36634327 PMC9846582

[B23] Kazemi A. Azimian J. Mafi M. Allen K. A. Motalebi S. A. (2021). Caregiver burden and coping strategies in caregivers of older patients with stroke. BMC Psychol. 9, 51. 10.1186/s40359-021-00556-z 33794995 PMC8017750

[B24] Krishnamurthi R. V. Ikeda T. Feigin V. L. (2020). Global, regional and country-specific burden of ischaemic stroke, intracerebral haemorrhage and subarachnoid haemorrhage: a systematic analysis of the global burden of disease study 2017. Neuroepidemiology. 54, 171–179. 10.1159/000506396 32079017

[B25] Kumar A. Yadav A. K. Singh V. K. Pathak A. Chaurasia R. N. Mishra V. N.. (2022). Caregiver burden in caregivers of stroke survivors: a hospital-based study. Ann. Indian Acad. Neurol. 25, 1092–1098. 10.4103/aian.aian_318_22 36911438 PMC9996526

[B26] Kumar R. Kaur S. Reddemma K. (2016). Burden, its predictors and quality of life in caregivers of stroke survivors at rural community, Punjab, India. J. Neurol. Neurorehabil. Res. 1, 1–7. 10.35841/neurology-neurorehabilitation.1.1.3-9

[B27] Larson J. Franzén-Dahlin A. Billing E. von Arbin M. Murray V. Wredling R.. (2008). The impact of gender regarding psychological well-being and general life situation among spouses of stroke patients during the first year after the patients' stroke event: a longitudinal study. Int. J. Nurs. Stud. 45, 257–265. 10.1016/j.ijnurstu.2006.08.021 17046770

[B28] Le Danseur M. (2020). Stroke rehabilitation. Crit. Care Nurs. Clin. N. Am. 32, 97–108. 10.1016/j.cnc.2019.11.004 32014164

[B29] Lee E. Y. Sohn M. K. Lee J. M. Kim D. Y. Shin Y. I. Oh G. J.. (2022). Changes in long-term functional independence in patients with moderate and severe ischemic stroke: comparison of the responsiveness of the modified barthel index and the functional independence measure. Int. J. Environ. Res. Public Health 19:9612. 10.3390/ijerph19159612 35954971 PMC9367998

[B30] Liu C. H. Chen Y. J. Chen J. S. Fan C. W. Hsieh M. T. Lin C. Y.. (2022). Burdens on caregivers of patients with stroke during a pandemic: relationships with support satisfaction, psychological distress, and fear of COVID-19. BMC Geriatr. 22, 958. 10.1186/s12877-022-03675-3 36514006 PMC9745281

[B31] Liu Z. Heffernan C. Tan J. (2020). Caregiver burden: a concept analysis. Int. J. Nurs. Sci. 7, 438–445. 10.1016/j.ijnss.2020.07.012 33195757 PMC7644552

[B32] Lui M. H. Lee D. T. Greenwood N. Ross F. M. (2012). Informal stroke caregivers' self-appraised problem-solving abilities as a predictor of well-being and perceived social support. J. Clin. Nurs. 21, 232–242. 10.1111/j.1365-2702.2011.03742.x 21707806

[B33] Menon B. Salini P. Habeeba K. Conjeevaram J. Munisusmitha K. (2017). Female caregivers and stroke severity determines caregiver stress in stroke patients. Ann. Indian Acad. Neurol. 20, 418–424. 10.4103/aian.AIAN_203_17 29184350 PMC5682751

[B34] Owolabi M. O. Thrift A. G. Martins S. Johnson W. Pandian J. Abd-Allah F.. (2021). The state of stroke services across the globe: Report of World Stroke Organization-World Health Organization surveys. Int. J. Stroke. 16, 889–901. 10.1177/17474930211019568 33988062 PMC8800855

[B35] Pinquart M. Sörensen S. (2003). Associations of stressors and uplifts of caregiving with caregiver burden and depressive mood: a meta-analysis. J. Gerontol. Ser. B Psychol. Sci. Soc. Sci. 58, 112–128. 10.1093/geronb/58.2.P112 12646594

[B36] Pinquart M. Sörensen S. (2006). Gender differences in caregiver stressors, social resources, and health: an updated meta-analysis. J. Gerontol. Ser. B Psychol. Sci. Soc. Sci. 61, 33–45. 10.1093/geronb/61.1.P33 16399940

[B37] Roopchand-Martin S. Creary-Yan S. (2014). Level of caregiver burden in jamaican stroke caregivers and relationship between selected sociodemographic variables. West Indian Med. J. 63, 605–609. 10.7727/wimj.2013.060 25803375 PMC4663964

[B38] Sinha P. Mehta R. S. Parajuli P. Chaudhary P. Kushwaha R. P. (2022). Burden of care among primary caregivers' of spinal cord injury patients attending a tertiary care center in Eastern Nepal. Discover. Soc. Sci. Health 2, 12. 10.1007/s44155-022-00016-y

[B39] Tooth L. McKenna K. Barnett A. Prescott C. Murphy S. (2005). Caregiver burden, time spent caring and health status in the first 12 months following stroke. Brain Inj. 19, 963–974. 10.1080/02699050500110785 16263639

[B40] Van Exel N. Koopmanschap M. A. van den Berg B. Brouwer W. Van den Bos G. (2005). Burden of informal caregiving for stroke patients. Cerebrovasc. Dis. 19, 11–17. 10.1159/000081906 15528879

[B41] Visser-Meily A. Post M. van de Port I. Maas C. Forstberg-Wärleby G. Lindeman E.. (2009). Psychosocial functioning of spouses of patients with stroke from initial inpatient rehabilitation to 3 years poststroke: course and relations with coping strategies. Stroke 40, 1399–1404. 10.1161/STROKEAHA.108.516682 19095973

[B42] Wafa H. A. Wolfe C. D. A. Emmett E. Roth G. A. Johnson C. O. Wang Y.. (2020). Burden of stroke in europe: thirty-year projections of incidence, prevalence, deaths, and disability-adjusted life years. Stroke 51, 2418–2427. 10.1161/STROKEAHA.120.029606 32646325 PMC7382540

[B43] Wang Y. Tyagi S. Hoenig H. Lee K. E. Venketasubramanian N. Menon E.. (2021). Burden of informal care in stroke survivors and its determinants: a prospective observational study in an Asian setting. BMC Public Health 21, 1945. 10.1186/s12889-021-11991-3 34702247 PMC8547090

[B44] Yu H. Wang X. He R. Liang R. Zhou L. (2015). Measuring the caregiver burden of caring for community-residing people with Alzheimer's Disease. PLoS ONE 10, e0132168. 10.1371/journal.pone.0132168 26154626 PMC4496054

[B45] Yue L. Jia C. Hu B. Zhang Z. Bai M. Wang S.. (2022). Caregiving stress among family caregivers of older adults living with disabilities in China. Geriatr. Nurs. 47, 226–231. 10.1016/j.gerinurse.2022.07.017 35987148

[B46] Zhu W. Jiang Y. A. (2018). Meta-analytic study of predictors for informal caregiver burden in patients with stroke. J. Stroke Cerebrovasc. Dis. 27, 3636–3646. 10.1016/j.jstrokecerebrovasdis.2018.08.037 30268368

